# HDAC2 targeting stabilizes the CoREST complex in renal tubular cells and protects against renal ischemia/reperfusion injury

**DOI:** 10.1038/s41598-021-88242-3

**Published:** 2021-04-27

**Authors:** David D. Aufhauser, Paul Hernandez, Seth J. Concors, Ciaran O’Brien, Zhonglin Wang, Douglas R. Murken, Arabinda Samanta, Ulf H. Beier, Lauren Krumeich, Tricia R. Bhatti, Yanfeng Wang, Guanghui Ge, Liqing Wang, Shayan Cheraghlou, Florence F. Wagner, Edward B. Holson, Jay H. Kalin, Philip A. Cole, Wayne W. Hancock, Matthew H. Levine

**Affiliations:** 1 Department of Surgery, University of Wisconsin, Madison, WI USA; 2 Department of Surgery, University of Pennsylvania, Philadelphia, PA USA; 3 Department of Pathology and Laboratory Medicine, Children’s Hospital of Philadelphia, Philadelphia, PA USA; 4 Division of Nephrology, Department of Pediatrics, Children’s Hospital of Philadelphia and University of Pennsylvania, Philadelphia, PA USA; 5 School of Medicine, Yale University, New Haven, CT USA; 6 Stanley Center for Psychiatric Research, Broad Institute of Harvard and MIT, Cambridge, MA USA; 7 Division of Genetics, Departments of Medicine and Biological Chemistry and Molecular Pharmacology, Harvard Medical School and Brigham and Women’s Hospital, Boston, MA USA; 8 Department of Pathology and Laboratory Medicine, University of Pennsylvania, Philadelphia, PA USA; 9 Department of Surgery, Children’s Hospital of Philadelphia, Philadelphia, PA USA

**Keywords:** Acute kidney injury, Drug discovery and development

## Abstract

Histone/protein deacetylases (HDAC) 1 and 2 are typically viewed as structurally and functionally similar enzymes present within various co-regulatory complexes. We tested differential effects of these isoforms in renal ischemia reperfusion injury (IRI) using inducible knockout mice and found no significant change in ischemic tolerance with HDAC1 deletion, but mitigation of ischemic injury with HDAC2 deletion. Restriction of HDAC2 deletion to the kidney via transplantation or PAX8-controlled proximal renal tubule-specific Cre resulted in renal IRI protection. Pharmacologic inhibition of HDAC2 increased histone acetylation in the kidney but did not extend renal protection. Protein analysis demonstrated increased HDAC1-associated CoREST protein in HDAC2-/- versus WT cells, suggesting that in the absence of HDAC2, increased CoREST complex occupancy of HDAC1 can stabilize this complex. In vivo administration of a CoREST inhibitor exacerbated renal injury in WT mice and eliminated the benefit of HDAC2 deletion. Gene expression analysis of endothelin showed decreased endothelin levels in HDAC2 deletion. These data demonstrate that contrasting effects of HDAC1 and 2 on CoREST complex stability within renal tubules can affect outcomes of renal IRI and implicate endothelin as a potential downstream mediator.

## Introduction

Renal ischemia/reperfusion injury (IRI) is a common medical problem that contributes to acute kidney injury (AKI) in diverse clinical settings, including cardiovascular surgery, sepsis, trauma, and renal transplantation, and leads to increased costs, healthcare utilization, morbidity, and mortality^1–7^. In kidney transplantation, renal IRI is manifested as delayed graft function and affects up to 30% of transplant recipients^7–9^. Specific pharmacologic therapies for AKI do not exist.

Histone/protein deacetylases (HDAC) enzymes are a highly conserved family of proteins that regulate access of transcriptional machinery to promoter and enhancer sites on coiled chromosomal segments as a result of their regulation of histone acetylation. These ubiquitous enzymes also regulate the acetylation of > 1750 non-histone proteins, and form scaffolds for large, multi-molecular protein complexes^10–12^. HDAC proteins are active in a number of pathologic processes, including IRI, with pan-HDAC inhibitors showing benefit in reducing IRI in multiple tissues, including cardiac, cerebral, retinal, and renal ischemia^13–18^. Our previous work demonstrated the protective effects of a pan-HDAC inhibitor, Trichostatin-A, as well as a class I-specific HDAC inhibitor, MS-275, that inhibits HDAC1-3 but not HDAC8 in murine models of renal IRI^19,20^.

We now evaluated the individual roles of three class I HDAC enzymes (HDAC1, 2, and 3) during renal IRI as part of our ongoing efforts to develop therapies to mitigate functional impairment and renal fibrosis. HDAC1 and 2 have typically been viewed as functionally redundant due to their highly homologous structures^10^. Both proteins are located within the nucleus as constituents of nuclear coregulatory complexes (CoREST, NuRD and Sin3), extending their effects on gene expression beyond regulation of histone acetylation ^21–24^. In contrast, HDAC3 forms a canonical, deacetylase-dependent complex with NCoR1/NCoR2 and can be recruited to the nucleus by various transcription factors^25^, or can function via a non-canonical deacetylase-independent manner independently of NCoR1/NCoR2^26^. One relevant non-histone protein whose functions are regulated by class I HDACs is endothelin-1 (Edn1)^27^. Edn1 is a potent vasoconstrictor produced by endothelial cells and renal tubular epithelial cells and is known to mediate kidney injury and fibrosis^28–31^. The current studies explored the roles of class I HDACs during renal IRI and found that despite their structural homology these enzymes have contrasting actions that likely have therapeutic consequences for the management of AKI.

## Results

### Differential effects of IRI following HDAC1 versus HDAC2 deletion

Global germline deletion of HDAC1, 2 or 3 in mice leads to intrauterine or neonatal lethality^17,32^. Inducible, post-developmental HDAC deletion was therefore undertaken in mice bearing individual floxed HDAC genes and the CreER^T2^ transgene via tamoxifen injection^33^. Deletion was confirmed by Western blotting and immunoperoxidase staining of contralateral kidneys harvested during IRI experiments (Suppl. Fig. 1). Female mice were subjected to 28 min of warm IRI through unilateral microvascular clamping of the renal pedicle and contralateral nephrectomy under tight temperature control. This ischemic duration was selected after extensive calibration demonstrated reproducible severe but survivable renal injury among female, WT C57BL/6 (B6) mice^19^. Renal function was monitored by daily blood urea nitrogen (BUN) and creatinine (Cr) measurements for 4 days post-injury, and kidneys were collected for histologic examination and computerized fibrosis scoring with Sirius Red at 28 days post-op^19^. In experiments involving tamoxifen-induced gene deletion, WT B6 control mice were injected with tamoxifen on the same schedule as experimental mice.

**Figure 1:**
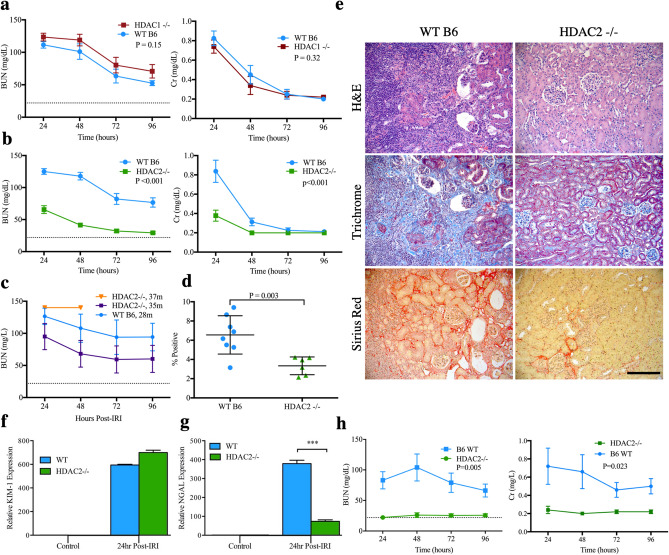
BUN and Cr after 28 min of warm IRI in WT female mice compared to (**a**) HDAC1-/- and (**b**) HDAC2-/- female mice. (**c**) BUN and Cr after extended IRI in HDAC2-/- female mice. HDAC2-/- mice exposed to 37 min of ischemia died from renal failure within 48 h of injury. Data are expressed as mean ± SEM (5 mice/group), and significance was determined with 2-way analysis of variance (ANOVA). Dotted line at BUN = 22 (and in subsequent panels of BUN data) reflects baseline BUN of WT C57BL/6 mice. (**d**) Computerized scoring of renal fibrosis at 28 days (Sirius Red staining); data are expressed as mean ± SD, and significance was determined by unpaired t-test, n = 8 WT, n = 6 H2-/- mice. (**e**) Representative hematoxylin and eosin (H&E), trichrome, and Sirius Red staining of kidneys collected 28 days after warm IRI from WT and HDAC2-/- mice, scale marker = 50 µM. Relative expression of (**f**) KIM-1 and (**g**) NGAL at baseline and 24 h following 28 min of warm IRI in WT B6 and HDAC2-/- kidneys; data are expressed as mean ± SD, and significance was determined by 2-way ANOVA, n = 3. (**h**) BUN and Cr following 15 min of warm IRI in HDAC2-/- and WT male mice; data are expressed as mean ± SEM (5 mice/group), and significance was determined by 2-way ANOVA.

Compared to WT B6 control mice, HDAC1-deficient mice (HDAC1-/-) did not show significant differences in renal function post-IRI (n = 10/group, Fig. 1a), nor did they have a significant increase in fibrosis at 28 days compared to WT B6, as assessed by quantitative Sirius Red staining (mean ± SEM of 6.3 ± 2.1% in HDAC1-/- vs. 6.0 ± 0.6% in WT, *p* = 0.84) (Suppl. Fig. 2a). In contrast, HDAC2-/- mice had a profound improvement in post-IRI renal function compared to controls (n = 17/group, *p* < 0.001, Fig. 1b). The magnitude of IRI protection in HDAC2-/- mice was further assessed by extending ischemic times beyond 28 min. HDAC2-/- mice tolerated up to 35 min of warm ischemia with similar impairment of renal function compared to control mice at 28 min of warm ischemia (n = 5/group; *p* = 0.26; Fig. 1c). HDAC2-/- mice receiving 28 min of warm ischemia had reduced renal fibrosis at 28 days (mean ± SEM of 3.3 ± 0.4% in HDAC2-/- vs. 6.6 ± 7.1% in WT, *p* = 0.003, Fig. 1d–e). While HDAC2-/- mice did not demonstrate a difference in expression of kidney injury marker 1 (Kim-1, *p* > 0.05, Fig. 1f), an adhesion molecule associated with post-injury regeneration^34–36^, they did demonstrate significantly decreased expression of neutrophil gelatinase-associated lipocalin (Ngal, *p* < 0.001, Fig. 1g), a biomarker of renal injury^37,38^. To assess whether the effects of HDAC2 depended on sex, male WT and HDAC2-/- mice were exposed to 15 min of warm IRI because of inferior ischemic tolerance previously observed in male mice^39^. HDAC2-deficient male mice demonstrated superior post-injury renal function compared to WT using this modified ischemia time (n = 5/group, *p* < 0.01) (Fig. 1h). Collectively, these studies showed that deletion of HDAC2 but not HDAC1 protected against IRI-induced renal injury.

### HDAC3 deletion in a transplant model

**Figure 2:**
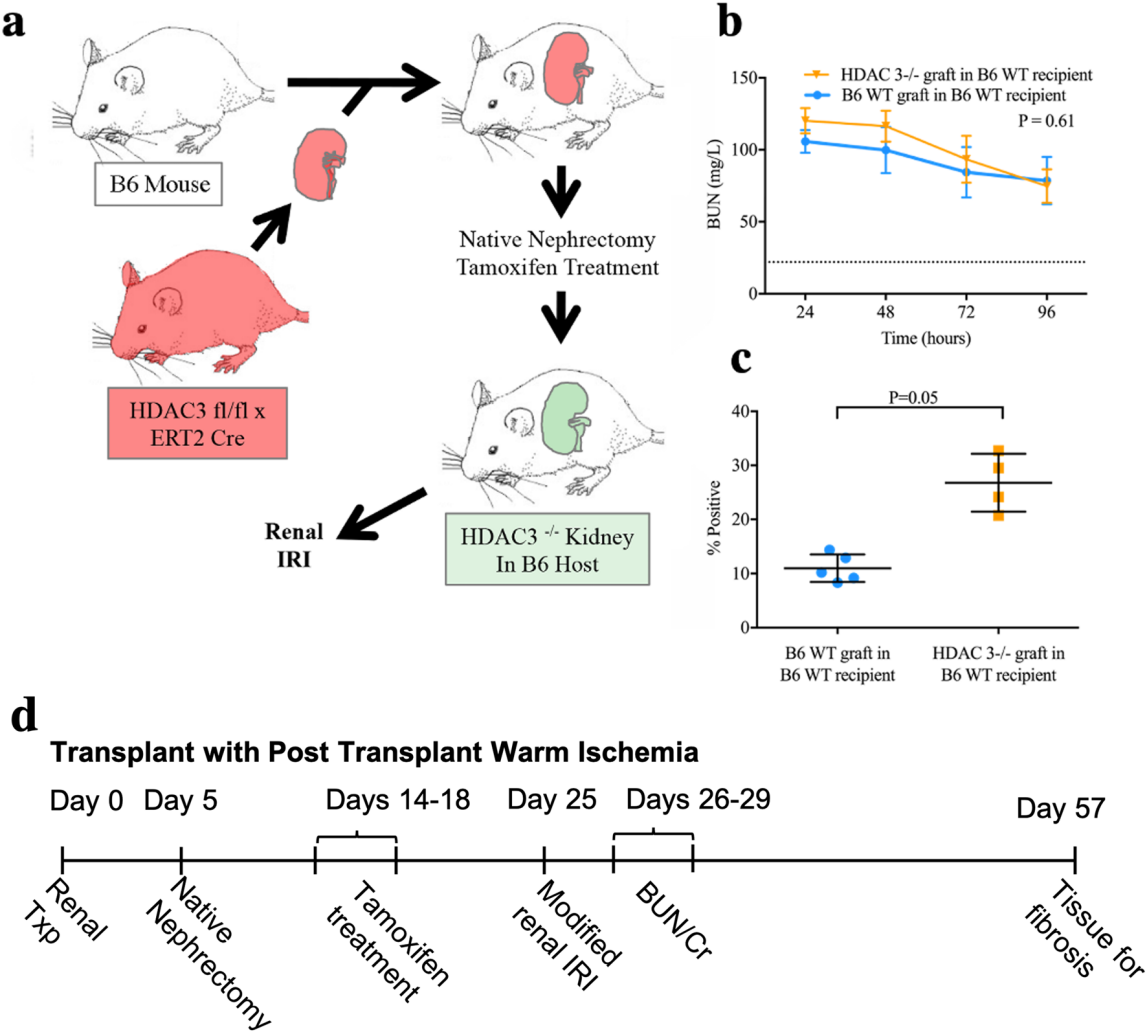
(**a**) Strategy for selective renal HDAC3 deletion prior to renal IRI: kidneys from B6 mice bearing floxed HDAC3 and CreER^T2^ transgenes were engrafted into WT B6 mice, followed by native nephrectomy and tamoxifen induction, and subsequently renal IRI. (**b**) BUN following 25 min of warm IRI in HDAC3-deleted renal isografts and WT renal isografts; data are expressed as mean ± SEM, and significance was determined by 2-way ANOVA (n = 5 mice/group). (**c**) Computerized scoring of renal fibrosis with Sirius Red staining at 28 days post-IRI in HDAC3- > WT (n = 4) and WT- > WT (n = 5) isografts; data were expressed as mean ± SD, and significance was determined by unpaired t-test. (**d**) Experimental timeline for renal transplant with IRI.

Mice with post-development whole-body deletion of HDAC3 developed lethargy and died by 2–3 weeks post-gene deletion, despite normal renal function. Complete postmortem examination and biochemical profiling of HDAC3-/- mice did not reveal a clear cause of death. This led us to test the effects of kidney-restricted HDAC3 deletion, by performing syngeneic kidney transplants between donor mice bearing floxed HDAC3 genes and the CreER^T2^ transgene and WT B6 recipient mice, or from WT B6 into WT B6 mice. Transplanted kidneys were allowed 2 week to recover from surgery before undergoing tamoxifen-induced gene deletion followed by standardized 25-min of warm ischemia by clamping the transplanted renal pedicle and undertaking bilateral native nephrectomy 2 week later (Fig. 2a). HDAC3-/- isografts exhibited similar renal injury (n = 5/group, *p* = 0.61, Fig. 2b) to WT B6 isografts, but had significantly increased fibrosis at 28 days (mean ± SEM of 26.8% ± 2.7% in HDAC3-/- isografts vs. 11.0% ± 1.1% in WT B6 isografts, *p* = 0.005, Fig. 2c); representative histologic data are shown in Suppl. Fig. 2b. Hence, renal deletion of HDAC3 did not protect against IRI.

### Beneficial effects of renal HDAC2 deletion

**Figure 3:**
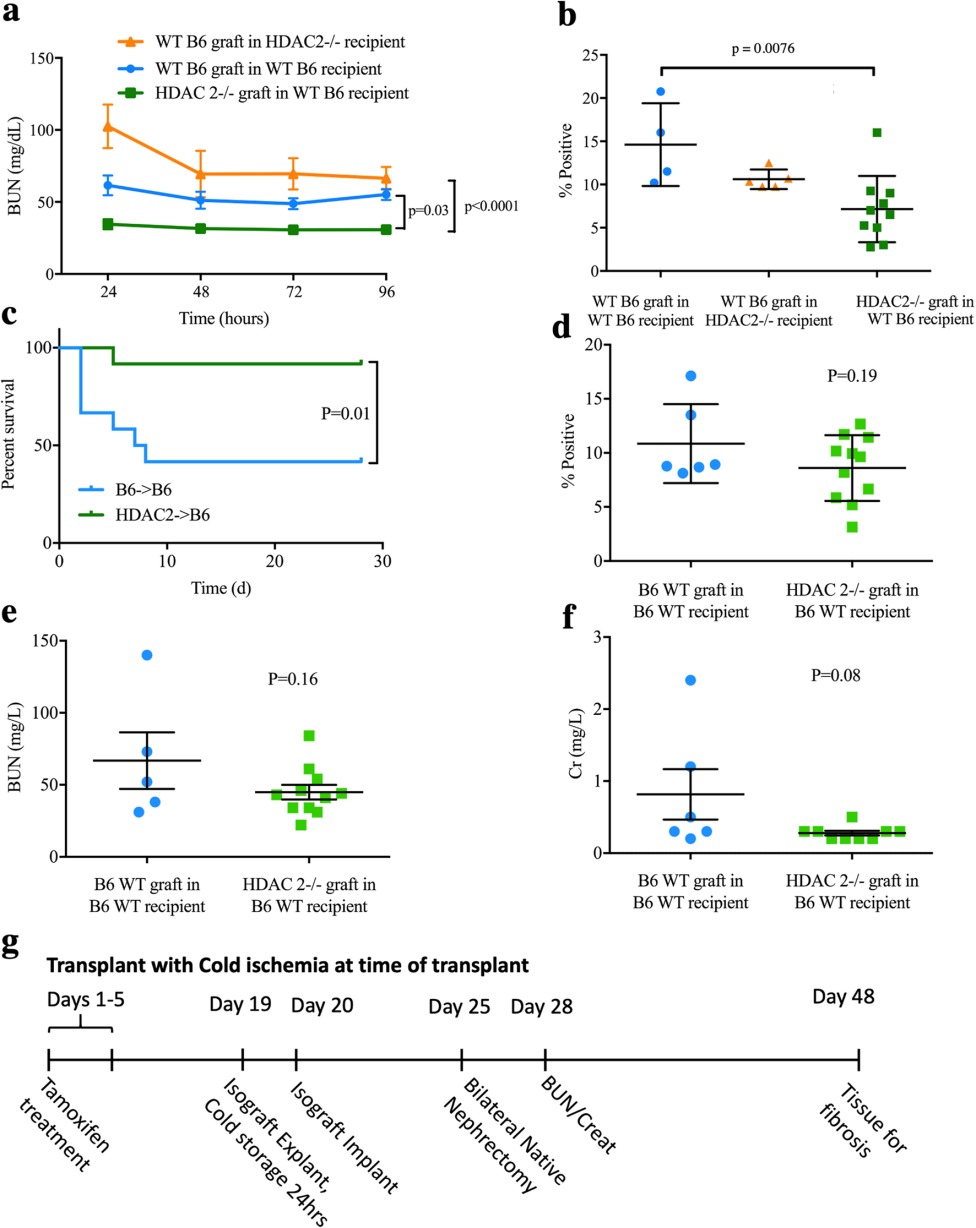
(**a**) BUN and (**b**) computerized scoring of renal fibrosis (Sirius Red stain) at 28 days in HDAC2-/- renal isografts in WT recipients (n = 10), WT B6 renal isografts in HDAC2-/- recipients (n = 5), and WT B6 renal isografts in WT recipients (n = 5). Data are expressed as mean ± SEM for (**a**) and mean ± SD for (**b**); significance was determined with 2-way ANOVA with Tukey’s post hoc test for (**a**), and with 1-way ANOVA with Tukey’s post hoc test for (**b**). (**c**) Survival after transplantation of HDAC2-/- or WT B6 renal isografts into a WT B6 recipient with 24-h cold ischemic times and bilateral native nephrectomy 5 days post-transplant; significance was determined by log-rank test (n = 12 mice/group at start). (**d**) Renal fibrosis with Sirius Red staining at 28 days among survivors; significance was determined using the unpaired t-test (n = 6 B6 WT in B6 WT and 11 HDAC2-/- in B6 WT). (**e**) BUN and (**f**) Cr at 8 days post-nephrectomy. (**g**) Experimental timeline for renal transplant with cold ischemia experiments.

We undertook renal transplantation to achieve tissue-restricted gene deletion and thereby assess whether the protection seen in HDAC2-/- mice was due to renal-intrinsic or renal-extrinsic factors. Kidneys were transplanted as isografts from mice bearing floxed HDAC2 genes and the CreER^T2^ transgene (HDAC2^fl/fl^) into WT mice, from WT B6 into HDAC2-/- mice, or from WT B6 into WT B6 mice. Gene deletion was induced by tamoxifen injection after recovery from transplantation, and as above, transplanted kidneys were subjected to 25 min of warm ischemia 2 week after gene deletion. WT B6 mice that received HDAC2^fl/fl^ isografts (n = 10) demonstrated significant improvement in warm IRI tolerance, with lower daily BUN levels over the 4 days post-ischemia, compared to HDAC2-/- mice that received WT B6 isografts (n = 5, *p* < 0.001) or WT B6 mice that received WT B6 isografts (n = 5, *p* = 0.03, Fig. 3a). WT B6 mice that received HDAC2^fl/fl^ isografts also showed a significant reduction in renal fibrosis at 28 days compared to the other groups (both *p* < 0.05, Fig. 3b). These data indicate that the site of protection induced by HDAC2 deficiency is intrinsic to the kidney.

### Cold ischemia protection by HDAC2 deletion

Protection from cold ischemia is particularly relevant in transplantation, where logistic and technical factors necessitate prolonged cold storage of organs. Accordingly, we tested the benefit of HDAC2 deletion by transplanting HDAC2-/- or WT B6 isografts subjected to cold ischemia at 4 °C while stored for 24 h in UW preservation solution. Mice underwent bilateral native nephrectomy 7 days post-transplant and renal function was monitored for 2 week. Recipients of HDAC2-/- isografts had superior survival compared to recipients of WT B6 isografts (n = 12 in each group at beginning of experiment, *p* = 0.01, Fig. 3c). Among survivors, there were no statistically significant differences in degree of fibrosis (*p* = 0.19, Fig. 3d) or altered renal function (*p* = 0.16, Fig. 3e, f). However, since more than 50% of the controls did not survive the peri-transplant period, the data in Fig. 3d–f reflect only the higher functioning survivors in that group; these metrics may understate the degree of long-term functional protection and fibrosis mitigation in HDAC2-/- isograft recipients.

### HDAC2 deletion reduces expression of endothelin and its receptors

**Figure 4:**
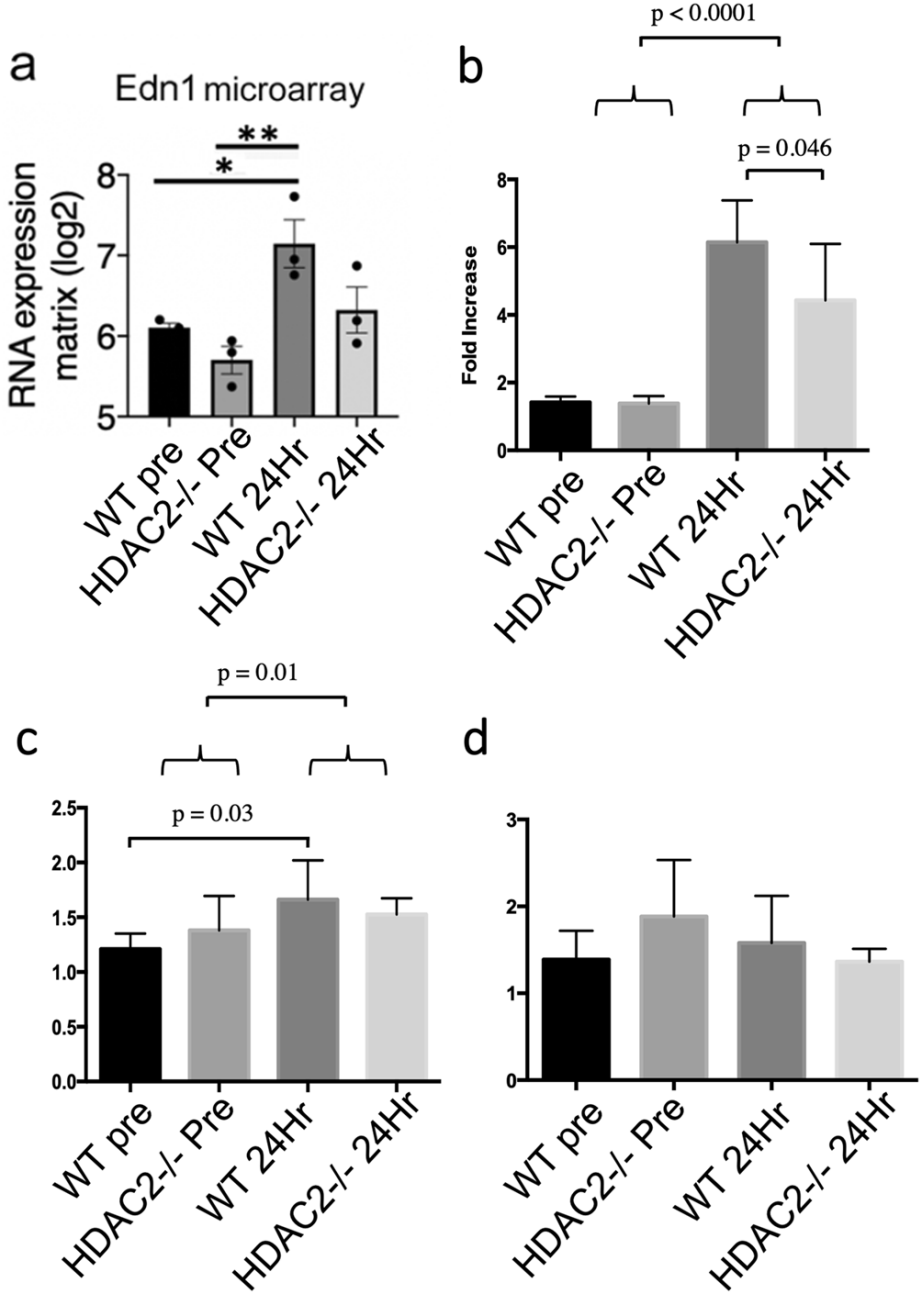
(**a**) Microarray gene expression levels in whole kidneys of wild type B6 mice and HDAC2-/- mice before and 4 h after IRI, n = 3, *p < 0.05. (**b**) qPCR expression levels comparing Edn1 levels in wild type C57BL/6 mice and HDAC2-/- mice before and 24 h after IRI, n = 6 (**c**) qPCR expression levels comparing Ednra levels in wild type B6 mice and HDAC2-/- mice before and 24 h after IRI, n = 6. (**d**) qPCR expression levels comparing Ednrb levels in wild type B6 mice and HDAC2-/- mice before and 24 h after IRI, n = 6.

Since HDAC2 regulates chromatin remodeling and gene expression, we utilized microarray gene expression analysis of whole kidneys at 4 h post-ischemia when in WT mice HDAC2 was activated as shown by phosphorylation at serine 394 (Suppl. Fig. S3a)^40^. While differential expression (twofold change, *p* < 0.05 ANOVA) affected only 0.05–3.68% of all transcripts (Suppl. Fig. S3b), changes included decreased heat shock and chemokine responses in IRI HDAC2-/- mice (Suppl. Fig. S3c). Notably, significantly increased levels of Edn1 were seen in ischemic WT but not HDAC2-/- mice (Fig. 4a). Edn1 is important to the progression of chronic kidney disease and renal scarring^28–31^, leading us to evaluate kidneys subjected to IRI at longer time-points for their expression of Edn1 and its receptors of relevance, Ednra and Ednrb. Compared to baseline, levels of Edn1 mRNA in WT and HDAC 2-/- kidneys were elevated several-fold at 24 h post-ischemia, though levels in WT mice were significantly higher than in HDAC2-/- mice (*p* = 0.046, Fig. 4b). In addition, Ednra mRNA expression were increased at 24 h post-ischemia in WT but not HDAC2-/- mice (Fig. 4c), whereas levels of Ednrb were unchanged (Fig. 4d). Hence, HDAC2 deletion leads to decreased upregulation of Edn1 and Ednra compared to their sequential upregulation in WT kidneys following renal IRI.

### Beneficial effects of renal tubule-specific HDAC2 deletion

**Figure 5:**
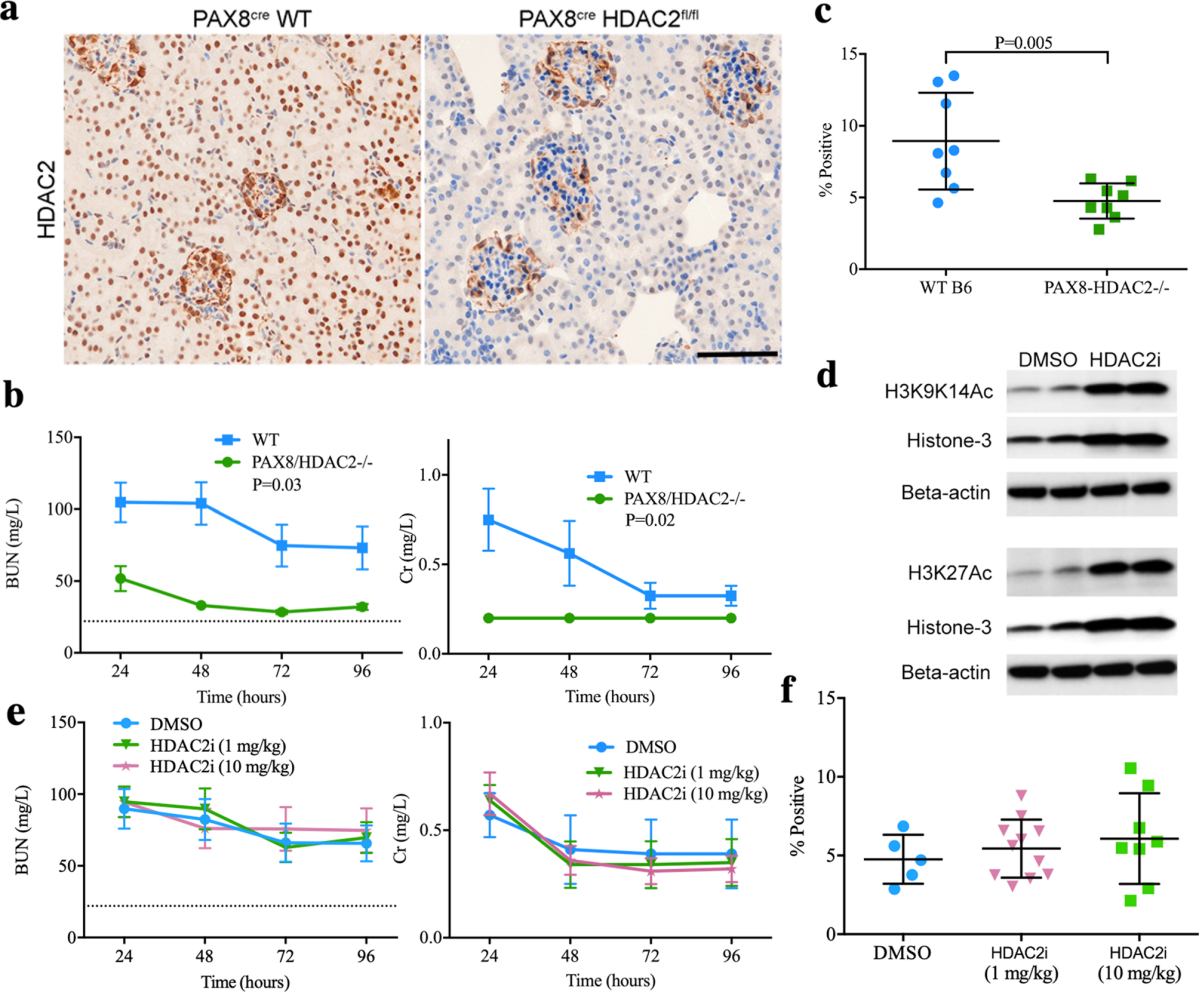
(**a**) Immunoperoxidase staining showed that compared to PAX8^cre^ controls, PAX8^cre^HDAC2^fl/fl^ mice showed decreased expression of HDAC2 within renal tubules on immunohistochemistry; hematoxylin counterstain, scale marker = 50 µM. (**b**) BUN and Cr following 28 min of warm IRI in PAX8^cre^HDAC2^f/fl^ (PAX8/HDAC2-/-) and PAX8^cre^ control mice; data are expressed as mean ± SEM, and significance was determined by 2-way ANOVA (n = 8 mice/group). (**c**) Renal fibrosis with Sirius Red staining at 28 days; significance was determined using the unpaired t-test. (**d**) Representative Western blots using nuclear extracts from kidneys from mice treated with HDAC2i (10 mg/kg/day) or vehicle (DMSO); data are representative of 3 independent experiments and original blots, including MS standards, are displayed in Suppl. Fig. 5. (**e**) BUN and Cr and (**f**) computerized scoring of renal fibrosis with Sirius Red staining at 28 days in WT B6 mice treated with low or high dose HDAC2i or vehicle (DMSO). Data are expressed as mean ± SEM for (**e**) and mean ± SD for (**f**), and significance was assessed by 2-way ANOVA for (**e**) and by unpaired t-test for (**f**); there were no significant differences for either dose of HDAC2i and vehicle (panels (**e**,**f**)).

To further investigate the tissue specificity of the benefit of HDAC2 deletion in renal ischemia, we generated mice with floxed HDAC2 and the PAX8-CreER^T2^ transgene, which leads to HDAC2 gene deletion within renal tubules upon Cre induction with tamoxifen^41^. Selective deletion of HDAC2 within renal tubules was confirmed by immunoperoxidase staining (Fig. 5a). When exposed to 28 min of warm, unilateral renal ischemia, PAX8^cre^HDAC2^fl/fl^ mice exhibited significant protection compared to WT mice both in terms of post-injury (Fig. 5b) BUN (*p* < 0.01) and Cr (*p* < 0.05). PAX8/HDAC2-/- mice also had reduced renal fibrosis at 28 days (Fig. 5c).

### Pharmacologic inhibition of HDAC2

To evaluate whether the effects of HDAC2 gene deletion could be replicated by pharmacologic inhibition, we administered BRD6688 (HDAC2i), a kinetically-selective HDAC2 enzymatic inhibitor^42^, or vehicle (DMSO), to WT B6 mice at 1 or 10 mg/kg/day at 16 h and 1 h prior to 28 min of warm IRI. Western blots of kidneys collected from mice treated with 10 mg/kg/day of HDAC2i demonstrated increased acetylation of histone-3 at multiple sites, indicating that the compound was active in renal tissue at this dose (Fig. 5d). However, HDAC2i did not significantly affect renal function (n = 10/group, Fig. 5e) or fibrosis formation (Fig. 5f), suggesting that the benefit of HDAC2 deletion may be independent of HDAC2 deacetylase catalytic activity.

### Impact of HDAC1 or 2 deletion on the CoREST complex

**Figure 6:**
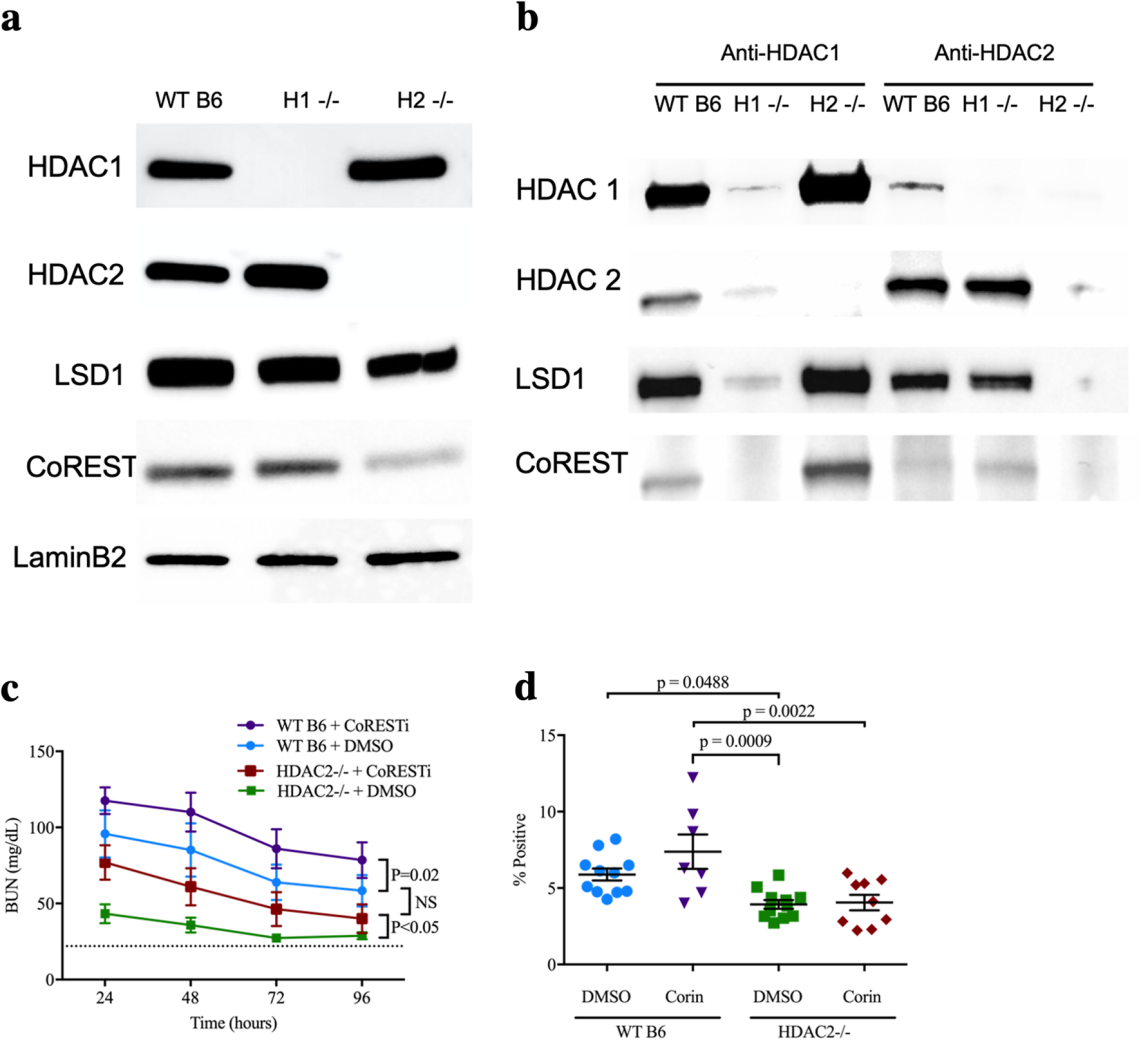
(**a**) Western blots using nuclear extracts from primary RTEC cultures from WT B6, HDAC1-/- (H1-/-) and HDAC2-/- mice (H2-/-); composite image displayed here, original blots, including MS standards, displayed in Suppl. Fig. 6a. (**b**) Western blots following immunoprecipitation with anti-HDAC1 or anti-HDAC2 antibody, using cells as described in panel (**a**). Composite image displayed here, original blots, including MS standards, are displayed in Suppl. Fig. 6b. (**c**) BUN and (**d**) computerized scoring of renal fibrosis with Sirius Red staining at 28 days in WT mice treated with CoRESTi (10 mg/kg at 16- and 1-h pre-ischemia) or vehicle (DMSO). Data are expressed as mean ± SEM for (**c**) and mean ± SD for (**d**). Significance was determined with 2-way ANOVA with Tukey’s post-hoc test.

HDAC1 and 2 contribute to several nuclear multimeric coregulatory complexes, including CoREST^21^. We hypothesized that changes in the structure or stability of these complexes might be responsible for the divergent renal IRI phenotypes seen with deletion of HDAC1 and 2, and the lack of benefit seen with inhibition of HDAC2 catalytic inhibition. To test this, we cultured renal tubular epithelial cells (RTEC) from WT B6, HDAC1-/-, and HDAC2-/- mice and analyzed the composition of the CoREST complex (Fig. 6a). HDAC2-/- RTEC had decreased total expression of CoREST compared to WT B6 or HDAC1-/- RTEC, and WT, HDAC1-/- and HDAC2-/- RTEC had equivalent expression of the CoREST constituent, LSD1 (KDM1a). Immunoprecipitation with anti-HDAC1 antibodies from corresponding cells showed increased HDAC1-associated CoREST and LSD1 in HDAC2-/- versus WT B6 (Fig. 6b). No equivalent increase in HDAC2-associated CoREST or LSD1 was seen upon immunoprecipitation with anti-HDAC2 antibodies in HDAC1-/- cells (Fig. 6b). These differences suggest the CoREST-HDAC1 complex may be more stable in the absence of HDAC2.

### In vivo CoREST inhibition

To evaluate further the role of CoREST in renal ischemia, we tested the effects of the CoRESTi, corin, in vivo^43^. Administration of corin or vehicle (DMSO), at 10 mg/kg/day at 16- and 1-h pre-ischemia, did not result in biochemical renal injury in either HDAC1-/- or HDAC2-/- mice. However, corresponding corin treatment did result in impaired renal IRI tolerance compared to DMSO-treated controls (n = 12/group, *p* ≤ 0.05, Fig. 6c). Treatment reversed the protective phenotype of HDAC2 deletion, with corin treated HDAC2-/- mice demonstrating similar renal impairment after injury as DMSO-treated WT mice, but did not significantly alter fibrosis at 28 days, possibly due to the limited duration of treatment (Fig. 6d). Hence, CoREST inhibition worsens the acute effects of renal IRI.

## Discussion

This study offers several novel insights into the functions of specific HDAC isoforms in renal IRI. Our findings challenge the concept that highly sequence-homologous HDAC1 and HDAC2 play functionally redundant roles in epigenetic regulation^10^. These experiments strongly suggest that the role these isoforms play in renal IRI is may be separable from their catalytic activity and highlight an impact of nuclear corepressor complex stability as a possible mechanism. Furthermore, these data link HDAC2 to the regulation of endothelin expression, a known mediator of renal injury^28–31^. The actions of HDAC2 are renal- and cell-specific and extend beyond suppression of fibrosis formation. These findings enhance our understanding of HDAC biology and provide a possible target for HDAC-based interventions beyond the HDAC catalytic site by altering HDAC occupancy in higher order corepressor complexes.

Our data indicate that the divergent functions of HDAC1 and HDAC2 in response to renal IRI include differential effects on the CoREST complex. When complexed with HDAC1 or HDAC2, LSD1 catalyzes demethylation of the H3K4me2 mark, promoting repression of target genes^44^. HDAC1 deacetylates LSD1 and promotes its demethylase activity^45^, and that CoREST promotes the nucleosome binding and demethylase activity of LSD1 while protecting it from proteasomal degradation^46^. The mechanism of renal protection in our studies appears linked to the enhanced stability and expression of the CoREST nuclear repressor complex in the absence of HDAC2, consistent with reversal of the HDAC2-/- phenotype occurring upon in vivo CoREST pharmacologic inhibition. This finding adds to literature suggesting that CoREST plays a significant role in mediating responses to ischemic injury and other forms of cellular stress. Noh et al. recently demonstrated in a model of ischemic stroke that pressor element-1 silencing transcriptional factor and CoREST play a crucial role in post-injury epigenetic remodeling^47^. In another study, CoREST was found to interact with Hsp70 and regulate cellular stress responses^48^. However, we have shown that Hsp70 modulation is not responsible for the effects of HDAC inhibition on renal ischemia^19^.

Our study also demonstrated that beyond their effects on acute injury, Class I HDAC can differentially affect IRI-induced renal fibrosis. Others have reported HDAC-mediated modulation of epithelial to mesenchymal cell transformation and resulting suppression of fibrosis in animal models of hypertensive nephropathy with the HDAC6i, Tubastatin-A; diabetic nephropathy with the pan-HDACi, TSA; and obstructive nephropathy with MS-275 and TSA^49–52^. Our findings, in a highly standardized renal IRI model, of HDAC3 deletion leading to increased fibrosis, HDAC1 deletion not significantly effecting fibrosis, and HDAC2 deletion resulting in decreased fibrosis, suggest that the anti-fibrotic effects of class I HDAC inhibition are fundamentally isoform-specific. The tissue-specificity of HDAC2 deletion points to these differences resulting from local tissue effects rather than broader influences on the immune system.

Given microarray data showing changes in endothelin levels confirmed by direct testing and in light of very recent data linking HDAC2 to regulation of Edn1 expression^27^, we hypothesized that Edn1 could be an important downstream mediator of HDAC2′s effects during renal IRI. Edn1 is produced by endothelial cells as well as renal tubular epithelial cells and is well known as a vasoconstrictor via its paracrine effects on vascular smooth muscle ET-A receptors. These effects play a role in hypertension, as well as mediating kidney damage independently of hypertension by increasing inflammation and kidney fibrosis^27,31,53^. Other groups have shown that endothelin is increased in response to renal IRI via a TNF-α mediated pathway^54^. Of particular interest, Zhang et al. showed, using a model of chronic kidney disease, that HDAC2 forms a complex with Dot1l, inhibiting its association with DNA, independent of HDAC2 catalytic activity^27^. Dot1l is a histone H3K79 methyltransferase that acts on the *Edn1* promotor to repress endothelin in renal tubular cells. In contrast to Zhang et al., our study used a validated model of renal IRI to link HDAC2 to both acute kidney injury and resulting fibrosis. Our findings that HDAC2 deletion, but not pharmacologic inhibition results in protection from renal IRI, as well as that HDAC2 deletion decreases endothelin expression, suggest that the interaction of HDAC2 with Dot1l is responsible for its effect in renal IRI.

Outside of HDAC biology, these findings hold clinical significance for their potential to facilitate new therapies for AKI, an exceedingly common and expensive medical problem that affects more than one million Americans per year, with costs in the billions of dollars annually^1–6^. AKI is frequent in multiple clinical situations, including renal transplantation, sepsis, trauma, and cardiovascular surgery and currently there are no targeted treatments available. Class I HDAC pathways offer a potential target for intervention to mitigate AKI in these diverse contexts. Although HDAC2 isoform-specific deacetylase inhibition did not replicate the profound IRI protection seen in HDAC2 deletion within the kidney, pharmacologic approaches to either inhibit HDAC2 occupancy in the CoREST complex, stabilize the CoREST complex itself, or alter Edn1 expression, as well as HDAC2 knockdown by antisense oligonucleotides, offer promising avenues to interrupt AKI development.

The study has several limitations. First, many of the in vivo transplant experiments described were conducted with relatively low *n* because of the complexity of the surgery involved. Despite the small group size, many of the results of these experiment were both statistically significant and supported with data from other complementary experiments. With repeats and various iterations of the HDAC2 experimentation, all experiments have led to positive impact on warm and cold IRI with HDAC2 deletion and the total number of experiments exceeds 50 animals, with multiple independent repeats. Second, the majority of the mouse experiments described were conducted in female mice. Sex differences in murine IRI are well established, and the focus on female mice was made in order to concentrate on a fully established and consistent model in our laboratory^39^. Sex differences in IRI tolerance dictate that the majority of experiments must be undertaken in mice of one sex or the other. We did confirm that HDAC2 deleted male also exhibited ischemic protection compared to WT male mice and believe that the effects described are independent of sex (Fig. 1h). Third, the broad nature of HDAC and CoREST actions complicate efforts to isolate downstream mechanisms responsible for the renal protection observed. Prior work suggests that Class I HDAC inhibition does not lead to anti-inflammatory modulation^19^. These data strongly suggest a renal-intrinsic and renal tubule-specific impact of HDAC2 deletion that would be most consistent with metabolic stabilization and anti-fibrotic activity, but further studies are necessary to delineate precise mechanisms. We have proposed Edn1 as a possible downstream mechanism, but complete delineation of the role of Edn1 in this process is under ongoing investigation, impaired by the ongoing pandemic.

In conclusion, we report on novel divergent function of HDAC1 and HDAC2 in response to renal IRI. HDAC2 targeting leads to profound protection from renal IRI through activity specific to kidney tissue, with data strongly suggesting an impact of the CoREST complex. Additionally, we have linked HDAC2 deletion with endothelin, a known mediator of renal injury. This pathway offers a promising target to better understand and treat AKI, a common clinical problem that is currently without options for pharmacologic therapy.

## Methods

### Animals

WT and UBC-Cre-ER^T2^ B6 mice (8–12 weeks old, 18–25 g) were purchased from The Jackson Laboratory. Production of HDAC1^fl/fl^, HDAC2^fl/fl^ and HDAC3^fl/fl^ mice were described previously^17,32^. Pax8-CreER^T2^ mice were obtained from the European Mouse Mutant Archive^41^. Cages of animals of equivalent age and the same shipment were chosen at random for experimental or control conditions. Conditional gene deletion was achieved by tamoxifen administration (100 mg/kg IP daily for 5 days, Sigma-Aldrich) with a 7-days recovery period after last administration before experimentation. Control mice received tamoxifen in the same manner.

### Pharmacologic agents

The HDAC2 selective inhibitor, BRD6688^42^, and the CoREST inhibitor, corin^43^, were > 95% pure and provided by their respective developers. The compounds were dissolved in DMSO, further dilution in phosphate-buffered saline, and 10 mg/kg was injected i.p. at 16 h and 1 h pre-ischemia.

### Warm IRI model

Warm IRI was performed as described previously^19^. Mice were anesthetized with pre-warmed pentobarbital sodium (65 mg/kg IP). Immediately after loss of a righting reflex, they were placed on a heated surgical pad (37 °C) in a temperature-controlled operative apparatus. Core body temperature was continuously measured throughout and maintained at 36.5 ± 0.5 °C. Using an operating microscope, an abdominal midline incision was made, and the left renal pedicle exposed and clamped for 28 min with a microvascular clip (Roboz Surgical Instrument Company). After the clamp was released, the right kidney was exposed and removed, the abdomen was closed, and animals were injected with 100 mL/kg of warm saline s.c. to assist in maintenance of hydration. Animals were kept in an incubator (37 °C) from the time of anesthetic administration until completely awake.

### BUN and Cr measurement

Whole blood BUN and whole blood Cr concentrations were assessed using i-STAT portable clinical analyzers with Chem8 + cartridges (Abbott Labs) that have a maximum BUN reading of 140 mg/dL and a minimum Cr reading of 0.2 mg/dL.

### Renal transplantation and subsequent warm IRI

Donor nephrectomy was performed under anesthesia with pentobarbital sodium (65 mg/kg, i.p.). The kidney was flushed with cold preservation (UW) solution and transplanted with donor renal artery, vein, and ureter anastomosed to recipient common iliac artery, common iliac vein, and bladder, with standardized time to reperfusion of 30 min during which the kidney was kept cool with iced gauze. Native kidneys were left in situ for 5 days and removed under inhalational isoflurane. Tamoxifen was administered (100 mg/kg/day, i.p., for 5 days) at 2 week post-transplant; 1 week after the last dose, mice underwent warm IRI experiments in the transplanted kidney, as outlined above, with microvascular clamping of the transplanted renal pedicle for 25 min.

### Cold IRI and subsequent transplantation

Conditional gene deletion was achieved through tamoxifen administration, as above. One week after last tamoxifen dose, renal transplantation was performed as outlined above, with a 24-h period of cold ischemic storage on ice. The native kidneys were left in situ for 5 days then removed under inhalational isoflurane anesthetic.

### Tissue collection and histopathology

Under terminal general anesthesia with pentobarbital sodium (65 mg/kg IP), the left kidney in warm IRI experiments or the transplanted kidney in cold IRI experiments was explanted at the indicated time, fixed in 10% neutral-buffered formalin and paraffin-embedded. Histologic sections (4 µm) were stained with hematoxylin and eosin (H&E), and Sirius Red. Slides were viewed with a Leica DM4 B microscope (Leica Microsystems) at 20 × and 40 × original magnification and images were captured with a Leica DFC480 camera. Sirius Red-stained sections were scanned using an Aperio ScanScope CS-0 slide scanner (Aperio Technologies). Whole slide digitized images were analyzed using the Aperio ImageScope software (version 12.2; Aperio Technologies) macro for color deconvolution version 9.0 optimized for Sirius Red cytochemical staining. Subcapsular areas were excluded to limit analysis to parenchymal changes.

### Immunoperoxidase

HDAC1 (Cell Signaling, 34589T) and HDAC2 (Sigma, SAB4501384-1004G) antibodies were used to stain formalin-fixed paraffin-embedded tissue slides. Staining was performed on a Bond Max automated staining system (Leica Biosystems) using the Bond Refine polymer staining kit (Leica Biosystems, DS9800). The standard protocol was followed with the exception that primary antibody incubation which was extended to 1 h at room temperature. HDAC1 and HDAC2 antibodies were used at 1:200, and 1:1000 dilutions respectively and antigen retrieval was performed with E2 (Leica Biosystems) retrieval solution for 20 min. Slides were rinsed, dehydrated, cover-slipped and digitally scanned at 20 × magnification on an Aperio CS-O slide scanner (Leica Biosystems) prior to evaluation.

### RNA isolation and quantitative PCR

RNA was extracted and purified using RNeasy Mini Kits (Qiagen) and reverse transcribed to cDNA using TaqMan reverse transcription reagents (Applied Biosystems). Quantitative PCR was performed using TaqMan Universal PCR Master Mix and gene-specific primer sets (NGAL: Mm01324470_m1; KIM-1: Mm00506686_m1; and 18S: Mm032928990_g1; Applied Biosystems). Samples were run on an ABI PRISM 7000 Sequence Detection System and compared using the ∆∆CT method.

### Primary RTEC culture

Under terminal general anesthesia, mouse kidneys were collected and the cortex was separated from the medulla, cut into small cubes, digested at 37 °C for 30 min with collagenase type 1 (Worthington Biochemical, 2 mg/mL), strained through a 100 μm nylon filter, and washed^55^. RTECs were grown in RPMI media supplemented with 10% FBS, EGF (Corning, 10 ng/L), penicillin (100 IU/mL), and streptomycin (100 μg/mL) at 37 °C with 5% CO_2_.

### Cell fractionation

To isolate nuclear protein extracts, cells were washed with cold PBS containing protease inhibitor mixture (Roche), lysed in a hypotonic buffer of 10 mM Hepes buffer (pH 7.6), 10 mM KCl, 1.5 mM MgCl2, 0.34 M sucrose, 10% glycerol, 1 mM DTT, and 0.1% Nonidet P-40 supplemented with 1 mM PMSF and protease inhibitor mixture, and incubated on ice for 10 min. Nuclei were extracted by centrifuging at 800 g for 5 min at 4 °C and lysed on ice with 20 mM Tris-Cl (pH 7.5), 300 mM NaCl, 0.5% NP40, 1 mM EDTA, 1 mM DTT, 1 mM PMSF and protease inhibitor mixture. Nuclear lysates were centrifuged at 17,000 g for 20 min and collected. Glycerol was added to lysates at a final concentration of 5%. Protein concentrations were estimated by bicinchoninic acid protein assay (Pierce).

### Immunoprecipitation and Western blotting

Nuclear lysates (150 µg) were immunoprecipitated with 3 µg of isotype-matched control or anti-HDAC1 monoclonal antibody (mAb) (Cell Signaling Technology) for 3 h at 4 °C, washed 4 times with washing buffer containing 20 mM Tris-Cl (pH: 7.5), 150 mM NaCl, 0.1% NP-40, 1 mM DTT, 1 mM EDTA, 1 mM PMSF and protease inhibitor mixture, and lysed by boiling with sample lysis buffer (BioRad Laboratories) containing β-mercaptoethanol. Western blotting was performed using anti-HDAC1 and anti-HDAC2 mAbs (Cell Signaling Technology), as well as polyclonal Abs directed against phosphorylated HDAC2 (Serine 394, Abcam), LSD1 (EMD Millipore), CoREST (EMD Millipore), and actin (Cell Signaling Technology).

### Microarray

RNA was isolated from frozen whole kidney tissue. Microarray experiments were performed using whole-mouse-genome oligoarrays (GeneChip™ Mouse Gene 2.0 ST arrays) and array data were analyzed using Transcriptome Analysis Console 4.0 software (ThermoFisher Scientific). Array data were subjected to robust multiarray average normalization. To assess differential gene expression, fold changes of up- and downregulated genes were calculated, and significance assessed using ANOVA with empirical Bayes parameter improvement (false-discovery adjusted *p* value of < 0.05).

### Data analysis

Data were analyzed using GraphPad Prism 8.0 software (GraphPad Software). Normally distributed data were displayed as mean ± standard error of the mean. Non-normally distributed data were with median and interquartile range. Measurements between two groups were performed with an unpaired Student-*t* test if normally distributed or Mann–Whitney *U* test if otherwise. Measurements between more than two group were performed using 1-way ANOVA with Tukey’s post hoc test. BUN and creatinine curves were compared using 2-way ANOVA with Tukey’s post hoc test as appropriate. Survival was assessed using a log-rank (Mantel–Cox) test.

### Study approval

All animal studies were approved by the Children’s Hospital of Philadelphia Institutional Animal Care and Use Committee (protocol 17-000954), performed at an AALAC accredited facility, and were conducted in accordance with all institutional policies and US federal guidelines. The study was carried out as much as possible in accordance with the ARRIVE guidelines (https://arriveguidelines.org/arrive-guidelines).

## Supplementary Information

Supplementary Information.

## Data Availability

The microarray dataset for this study can be found in the in the Gene Expression Omnibus database (http://www.ncbi.nlm.nih.gov/geo), under the accession number GEO: GSE131288.
