# Supplementary material for: HDAC2 targeting stabilizes the CoREST complex in renal tubular cells and protects against renal ischemia/reperfusion injury
Source: Sci Rep. 2021 Apr 27;11:9018. doi: 10.1038/s41598-021-88242-3 (PMC8079686; doi:10.1038/s41598-021-88242-3)
Supplement: Supplementary file 1 — Supplementary Information. [file 41598_2021_88242_MOESM1_ESM.pdf]

Supplemental Material

HDAC2 targeting stabilizes the CoREST complex in renal tubular cells and protects against renal ischemia/reperfusion injury

David D. Aufhauser Jr,[1] Paul Hernandez,[1] Seth J. Concors,[1] Ciaran O'Brien,[1] Zhonglin Wang,[1] Douglas R. Murken,[1] Arabinda Samanta,[2] Ulf H. Beier,[3] Lauren Krumeich,[1] Tricia R. Bhatti,[2] Yanfeng Wang,[1] Guanghui Ge,[1] Liqing Wang,[2] Shayan Cheraghlou,[4] Florence F. Wagner,[5] Edward B. Holson,[5] Jay H. Kalin,[6] Philip A. Cole.[6] Wayne W. Hancock,[2,7] & Matthew H. Levine[1,8]*

[1]Department of Surgery, University of Pennsylvania, Philadelphia, PA, USA. [2]Department of Pathology and Laboratory Medicine, Children's Hospital of Philadelphia, Philadelphia, PA, USA. [3]Division of Nephrology, Department of Pediatrics, Children's Hospital of Philadelphia and University of Pennsylvania, Philadelphia, PA, USA. [4]School of Medicine, Yale University, New Haven, CT, USA. [5]Stanley Center for Psychiatric Research, Broad Institute of Harvard and MIT, Cambridge, MA, USA. [6]Division of Genetics, Departments of Medicine and Biological Chemistry and Molecular Pharmacology, Harvard Medical School and Brigham and Women's Hospital, Boston, MA, USA. [7]Department of Pathology and Laboratory Medicine, University of Pennsylvania, Philadelphia, PA, USA. [8]Department of Surgery, Children's Hospital of Philadelphia, Philadelphia, PA, USA.

***Correspondence:** Matthew.Levine@pennmedicine.upenn.edu

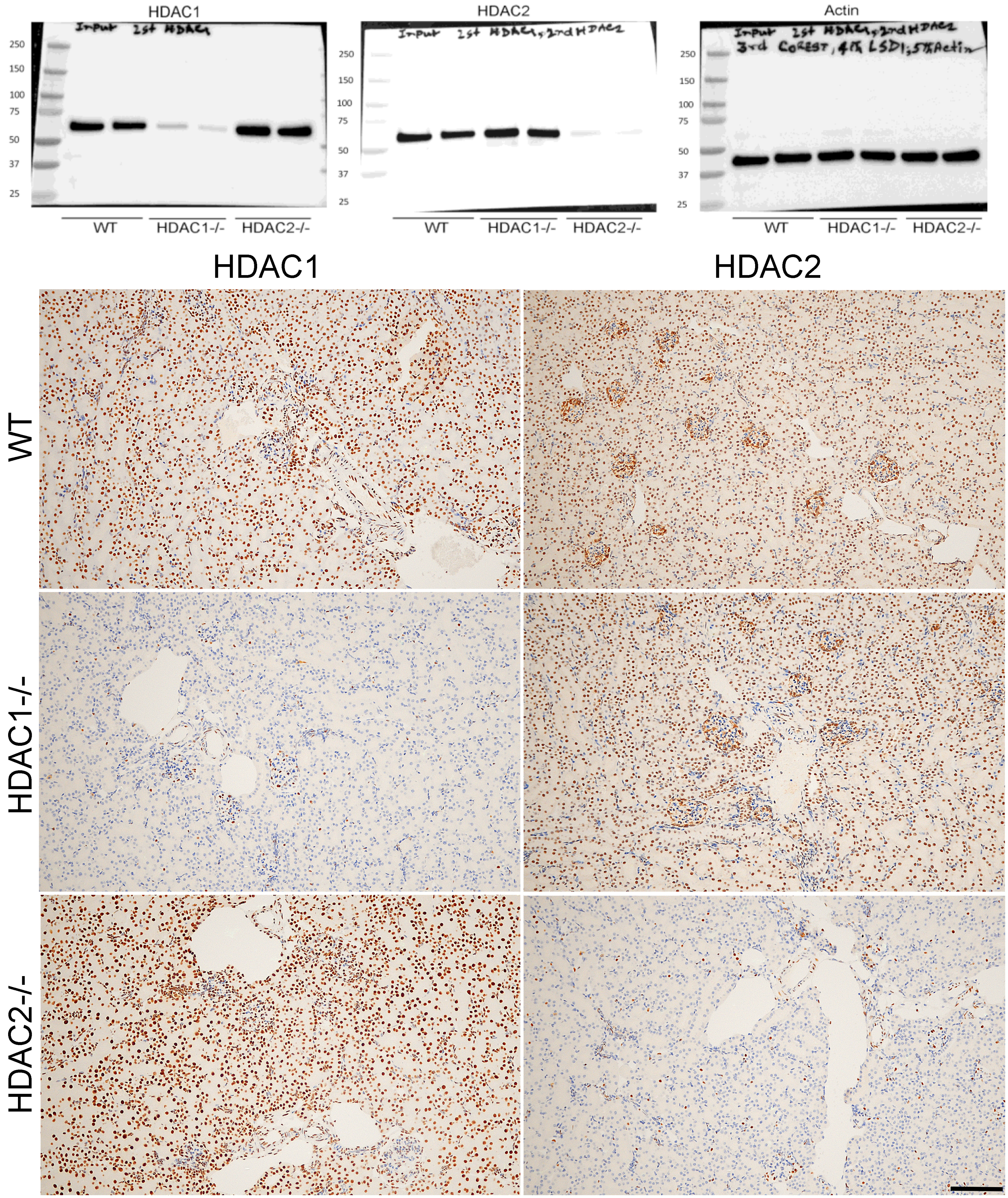

Suppl. Fig. 1. Western blot and representative immunoperoxidase staining of cortical portions of kidneys from WT B6, HDAC1-/- and HDAC2-/- mice demonstrating deletion of targeted proteins following tamoxifen treatment. Residual endogenous peroxidase positive RBC are seen within intertubular and glomerular capillaries of Hdac1-/- and Hdac2-/- kidney sections stained for HDAC1 and HDAC2, respectively. Paraffin sections, hematoxylin counterstain, scale bar = 50 µM.

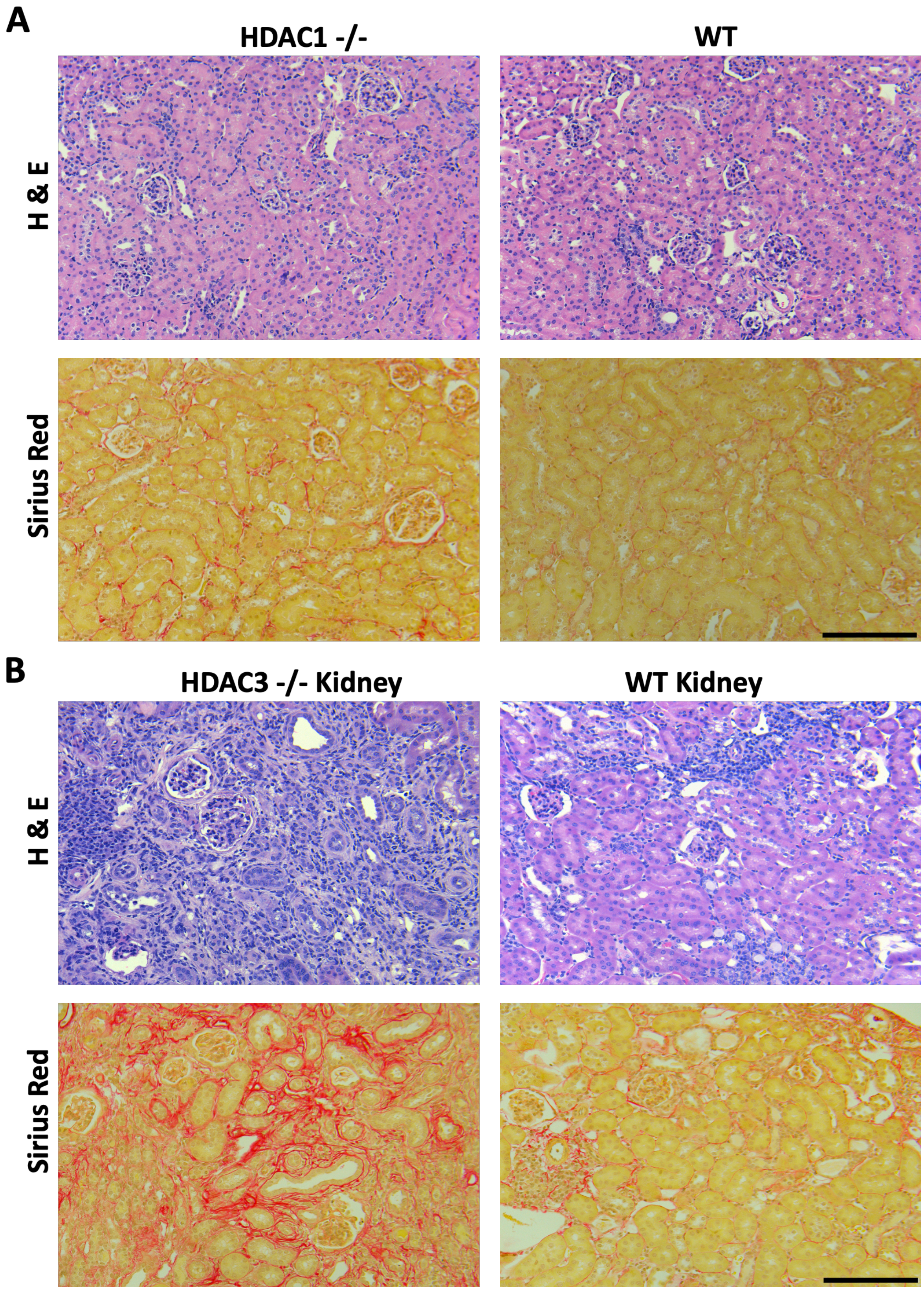

Suppl Fig. 2. (A) Representative histology from HDAC 1-/- kidneys following 28 minutes of renal IRI as compared to that of WT B6 mice and showing equivalent degrees of fibrosis. (B) Representative histology from HDAC3-/- kidneys transplanted into WT mice, following 25 minutes of renal IRI, as compared to WT kidneys transplanted into WT mice, showing increased fibrosis in the HDAC3-/- kidneys. Images obtained from slides scanned with Aperio ImageScope software (version 12.2; Aperio Technologies.)

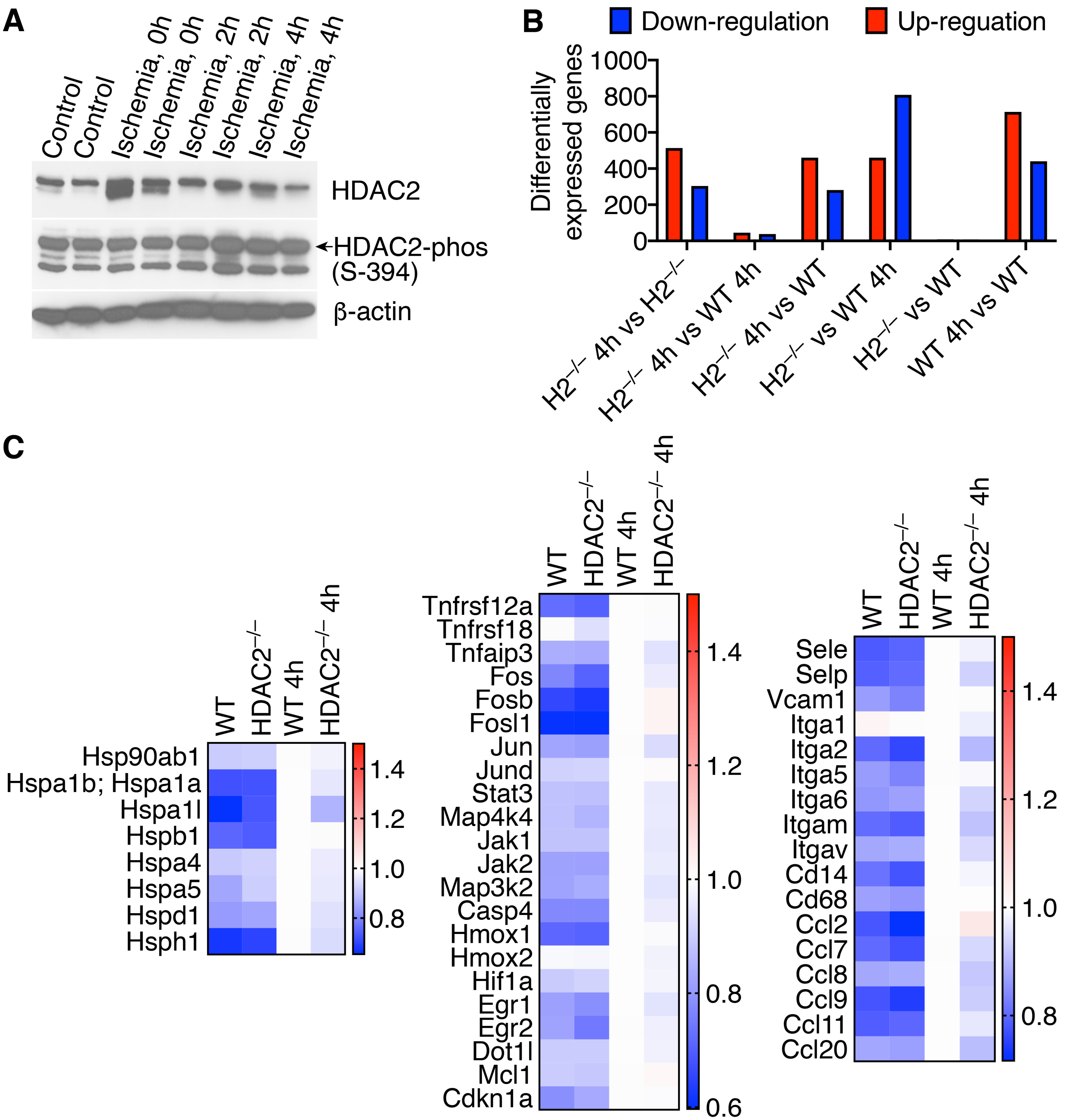

Suppl. Fig. 3. (A) Western blot of C57Bl/6 kidneys with and without warm renal IRI (full blots are shown in Suppl. Fig. 4). Data show 2 independent experiments. (B, C) GeneChip Mouse Gene 2.0 ST microarray of kidneys from WT and HDAC2$^{-/-}$ (H2$^{-/-}$) without or after 4 h of warm renal IRI. Data derived from 3 independent replicates per condition (B) Number of genes with significantly different ($p<0.05$ ANOVA) and ≥2-fold increased or decreased expression are shown for each pairing (out of 34,772 transcripts). (C) Selected heat maps showing mean RMA normalized and $\log_2$ transformed expression per group (n=3) normalized to C57BL/6 mice (WT) after 4 h of renal IRI. Abbreviations: Hsp, Heat shock protein; Tnfrsf, tumor necrosis factor receptor superfamily; Fos, FBJ osteosarcoma oncogene; Jun, jun proto oncogene; Stat, signal transducer and activator of transcription; Mapk, mitogen-activated protein kinase; Jak, janus kinase; Casp, caspase; Hmox, heme oxygenase; Hif, hypoxia inducible factor; Egr, early growth response; Mcl, myeloid cell leukemia sequence; cyclin-dependent kinase inhibitor; Sel, selectin; Vcam, vascular cell adhesion molecule; Itga, integrin alpha; Ccl, chemokine (C-C motif) ligand. Original blots to panel (A) in Suppl. Fig. 3.

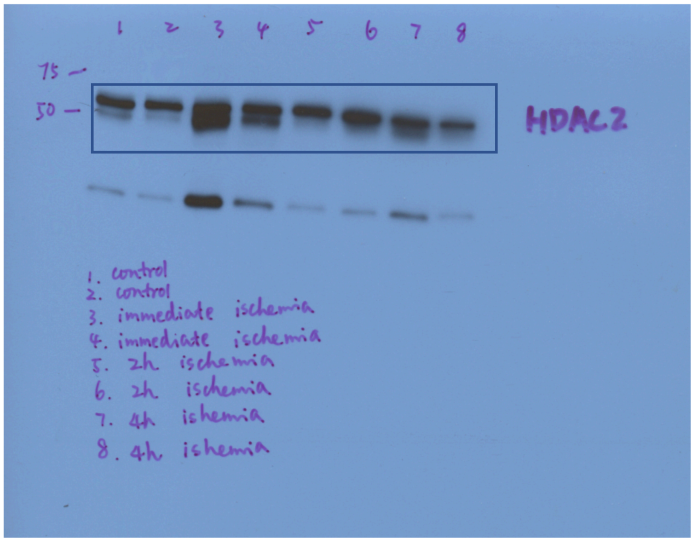

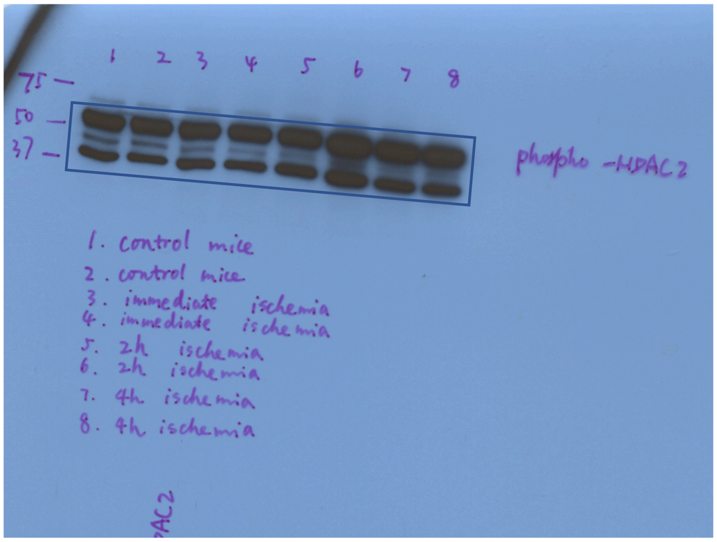

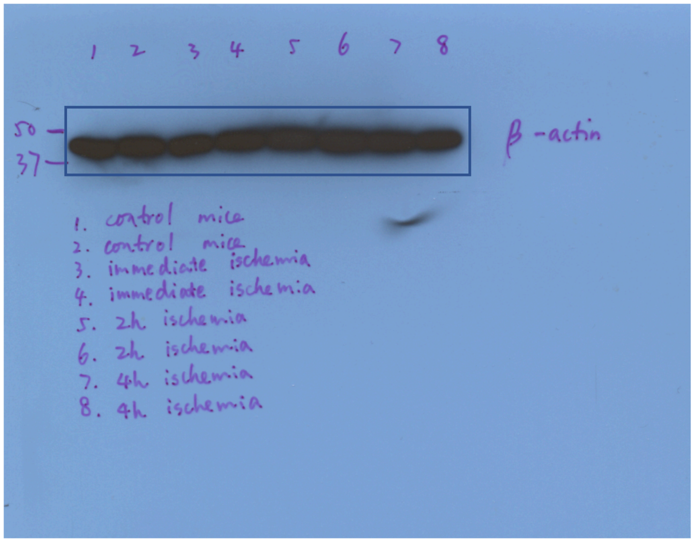

Suppl. Fig. S4. Original blots for Suppl. Fig. S3A.

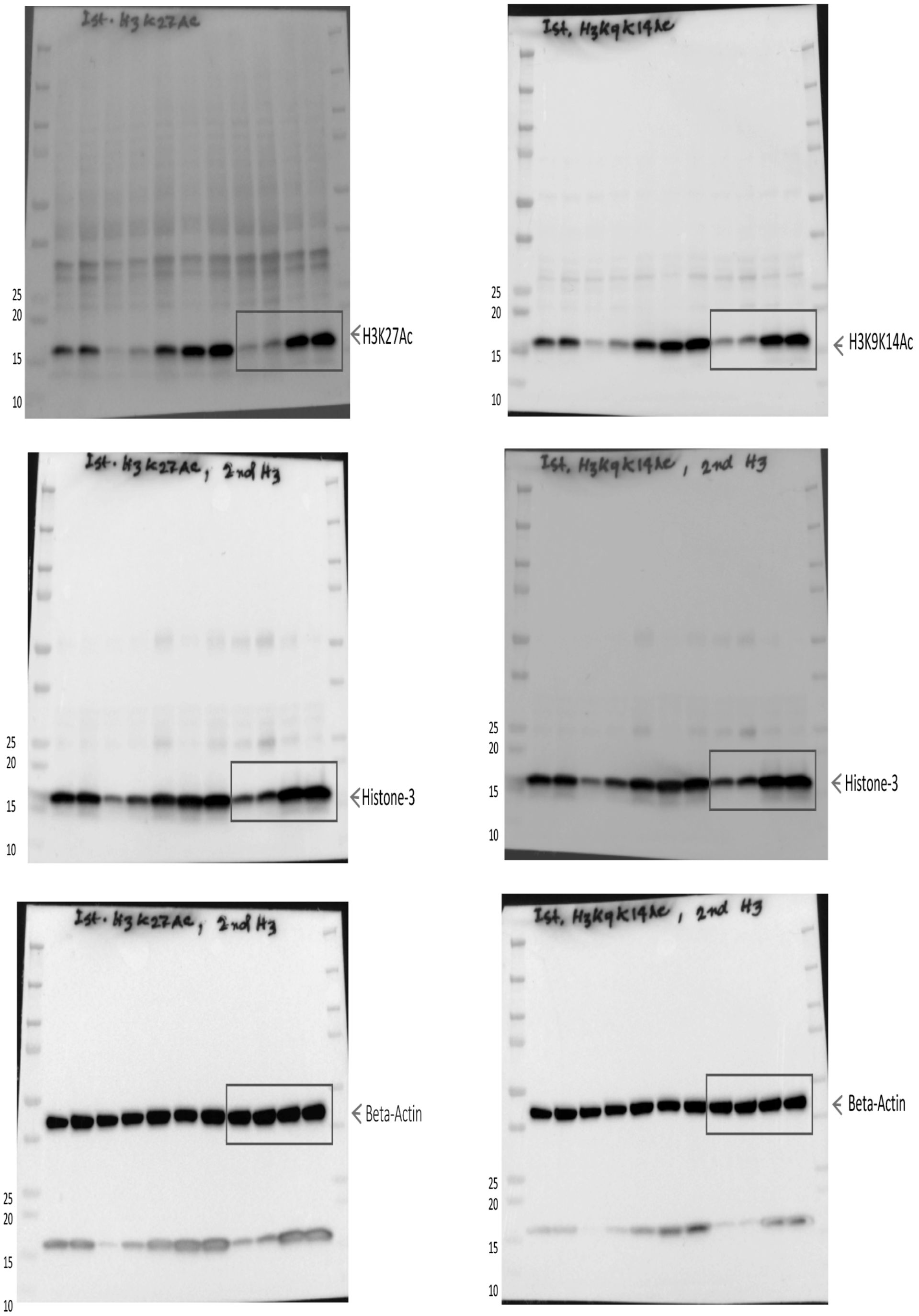

Suppl. Fig. 5. Original blots for Suppl. Fig. 5d. Western blots using nuclear extracts from kidneys from mice treated with DMSO (left 2 lanes of boxed area) or HDAC2i (10 mg/kg/d, right 2 lanes of boxed area); data are representative of 3 independent experiments. Gels were initially probed for H3K29Ac (left) or H3K9K14Ac (right), then Histone-3, and lastly for beta-actin.

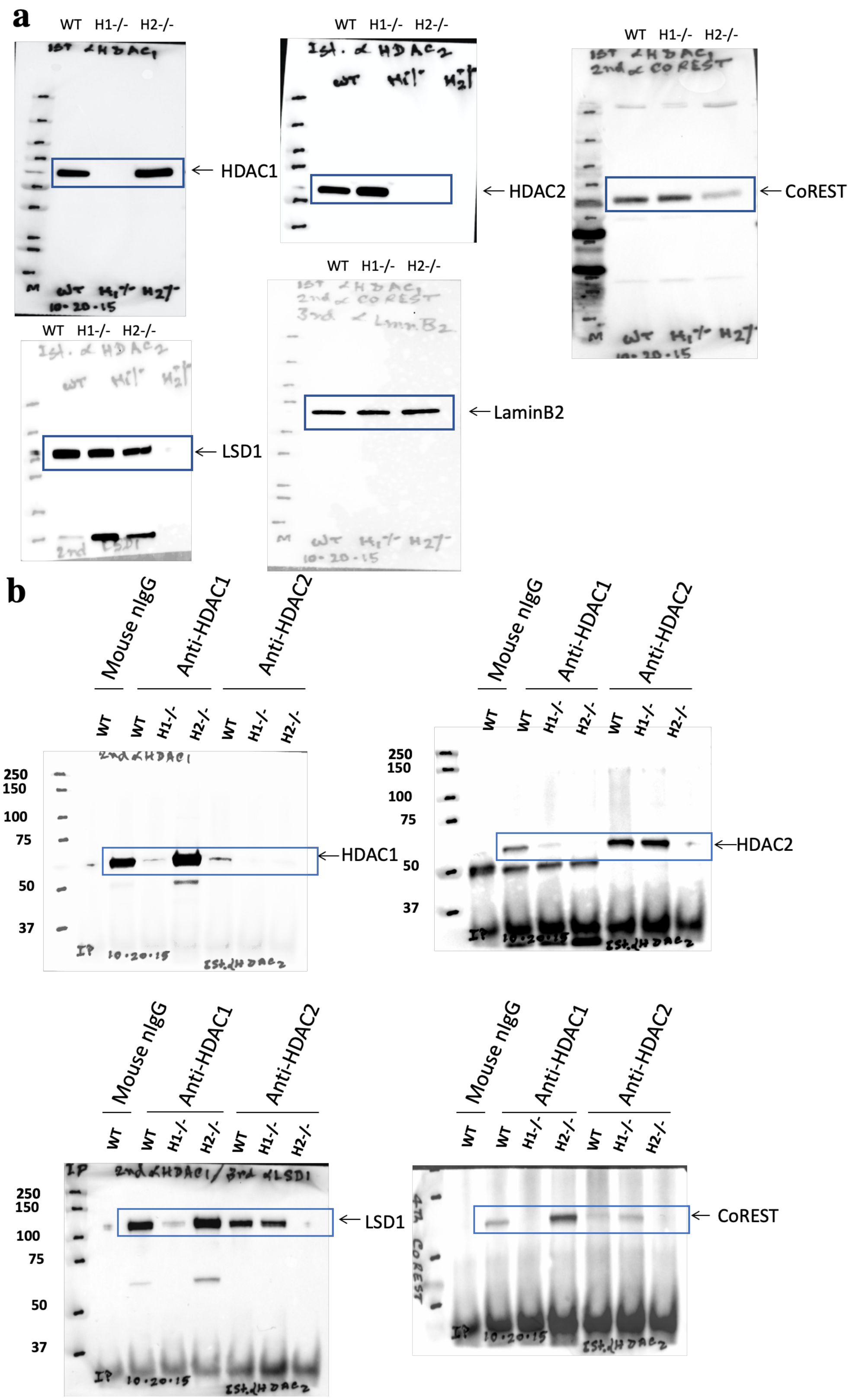

Suppl. Fig. 6. (a) Western blots using nuclear extracts from primary RTEC cultures from WT B6, HDAC1-/- and HDAC2-/- mice; (b) Western blots following immunoprecipitation with anti-HDAC1 antibody.
